# Effects of inspiratory muscle Pre-activation on 100 m sprint performance in physically active university students

**DOI:** 10.3389/fspor.2026.1840117

**Published:** 2026-07-20

**Authors:** Javier Carrión, Stalin Javier Caiza Lema, Samira Anahí Masaquiza Sanguil, Maria Augusta Latta Sánchez, Andrea Carolina Peñafiel Luna, Paúl Adrián Arias Córdova, Angela Priscila Campos Moposita, Josselyn Gabriela Bonilla Ayala, Martha Montalvan, Raynier Zambrano-Villacrés

**Affiliations:** 1 Department of Chemistry, Universidad Técnica Particular de Loja, Loja, Ecuador; 2 Carrera de Fisioterapia, Facultad Ciencias de la Salud, Universidad Técnica de Ambato, Ambato, Ecuador; 3 Escuela de Medicina, Universidad Espíritu Santo, Samborondón, Ecuador; 4 Facultad de Ciencias de la Salud y Desarrollo Humano, Universidad ECOTEC, Samborondón, Ecuador; 5 Escuela de Posgrado, Doctorado en Ciencias Biomédicas, Instituto Universitario Italiano de Rosario (IUNIR), Rosario, Argentina

**Keywords:** anaerobic performance, inspiratory muscle pre-activation, physically active university students, respiratory, sprint

## Abstract

**Introduction:**

Inspiratory muscle function may influence high-intensity exercise through mechanical stabilization and fatigue-related mechanisms. Inspiratory muscle pre-activation has been proposed as an ergogenic strategy; however, its effectiveness in short-duration sprint performance remains unclear. This study examined the acute effects of inspiratory muscle pre-activation on 100 m sprint performance in physically active university students.

**Methodology:**

A within-subject, quasi-experimental design was employed with 25 participants performing two warm-up conditions: peripheral muscle activation (PMA) and PMA plus inspiratory muscle activation (IMA). The inspiratory protocol consisted of two sets of 15 breaths at 40% of maximal inspiratory pressure. Sprint performance was assessed using a 100 m maximal effort, and differences were analyzed using the Wilcoxon signed-rank test (*p* ≤ 0.05).

**Results:**

Mean sprint times were 12.92 ± 2.12 s for PMA and 12.96 ± 2.10 s for IMA. Although a statistically significant difference was observed (*p* < 0.05), the magnitude of change (<0.05 s) was trivial. Sex-stratified descriptive values showed the same trivial pattern in women and men, although the sample size and unequal sex distribution precluded sex-based inference. No meaningful correlations were found between inspiratory strength and sprint performance.

**Discussion:**

The findings indicate that inspiratory muscle pre-activation did not produce a practically meaningful performance benefit in short-duration sprinting. The limited role of respiratory factors in the 100 m sprint and the dominance of neuromuscular determinants likely explain the absence of performance enhancement.

**Conclusion:**

Inspiratory muscle pre-activation did not produce a practically meaningful improvement in 100 m sprint performance in physically active individuals. It may be included within a broader warm-up routine, but the present data do not support its use as a primary strategy to improve 100 m sprint performance.

## 1 Introduction

The 100 m sprint is a short-duration, high-intensity event in which performance depends mainly on rapid force production, efficient mechanical power transfer, sprint mechanics, intermuscular coordination, and the capacity to apply large horizontal and vertical ground reaction forces during acceleration and maximal velocity phases (1–5). Energy supply is dominated by phosphagen metabolism, with limited oxidative contribution because of the brief duration of the event (6, 7). For this reason, sprint research and warm-up strategies have commonly focused on lower-limb muscle function, technique, muscle temperature, and neuromuscular readiness (3, 4, 8). Respiratory factors are not primary determinants of 100 m performance, yet the inspiratory muscles may contribute indirectly during maximal running through ventilation, trunk stabilization, and regulation of intra-abdominal pressure (9–16). This stabilizing role may be relevant during the start and early acceleration phases, where sprint posture, lumbopelvic control, and acceleration mechanics support force transmission under high mechanical demand (12, 16–19).

Respiratory muscle function may also influence high-intensity exercise through fatigue-related mechanisms (20–23). During strenuous exercise, increased work of breathing can activate the inspiratory muscle metaboreflex, which has been associated with altered blood flow distribution toward respiratory muscles and away from locomotor muscles (24–27). This mechanism is more relevant to prolonged or repeated high-intensity exercise than to a single 100 m sprint, so its role in short sprint performance should be interpreted cautiously (28–31). Even so, inspiratory muscle training and acute inspiratory muscle warm-up have attracted interest as strategies to improve respiratory muscle readiness before exercise (32–35). Acute inspiratory muscle pre-activation usually consists of resisted inspirations prescribed relative to maximal inspiratory pressure (PImax), with the aim of preparing the inspiratory muscles before competition or testing (35).

The mechanisms proposed for inspiratory muscle pre-activation include increased neural drive, improved coordination between respiration and trunk stabilization, reduced respiratory muscle strain, and possible potentiation-like effects after acute respiratory loading (33, 36–39). Evidence suggests that these effects are task-specific (40). Positive findings have been reported in endurance running, swimming, rowing, and intermittent sport contexts (2, 40–43), whereas other studies have shown trivial or inconsistent effects depending on exercise modality, training status, protocol dose, and outcome measure (32–35, 44). This variability indicates that inspiratory pre-activation should not be assumed to improve all forms of exercise performance.

Evidence remains limited for isolated short-distance sprinting (45). Most previous studies have examined endurance-based tasks, repeated-sprint efforts, or sport-specific skills in team sports rather than a single maximal 100 m sprint (20, 46–48). This distinction is relevant because the physiological demands of a 100 m sprint differ from middle-distance, intermittent, or prolonged exercise. Therefore, evidence obtained from endurance or repeated-effort tasks should not be directly extrapolated to a single sprint dominated by neuromuscular, technical, and mechanical determinants. Physically active university students represent an applied population capable of maximal sprint efforts but without the technical refinement or respiratory-specific adaptations expected in trained sprinters (24, 28, 49). In this context, assessing whether a brief, low-cost inspiratory pre-activation protocol can modify sprint performance has practical value for coaches, therapists, and exercise professionals. Protocol dose also remains relevant, since previous studies have used different combinations of inspiratory load, number of breaths, sets, and recovery intervals (50–52). Given that sprint performance is sensitive to very small time differences, the practical magnitude of any change must be interpreted alongside statistical significance (4, 27, 53).

The present study examined the acute effects of adding inspiratory muscle pre-activation to a conventional warm-up on 100 m sprint performance in physically active university students. Sprint performance after peripheral muscle activation alone was compared with performance after the same warm-up supplemented with inspiratory muscle activation. Based on the possible indirect mechanical and physiological contributions of the inspiratory muscles, it was hypothesized that inspiratory muscle pre-activation might produce a small but uncertain change in sprint performance.

## 2 Materials and methods

### 2.1 Design and ethical considerations

A quantitative, within-subject, quasi-experimental repeated-measures design was used to examine the acute effects of inspiratory muscle pre-activation on 100 m sprint performance in physically active university students. Each participant completed two experimental warm-up conditions in separate testing sessions: a peripheral muscle activation protocol (PMA) and an inspiratory muscle activation protocol (IMA). Because all participants were exposed to both conditions, each individual served as his or her own control, thereby reducing between-subject variability and increasing sensitivity for detecting small acute changes in sprint performance. This design was considered appropriate because the main outcome, sprint time over a standardized 100 m distance, is strongly influenced by individual characteristics such as sex, anthropometry, fitness level, coordination, and prior sprint experience.

Data collection was conducted in Ecuador between June and July 2025, whereas data analysis and manuscript development took place between August and October 2025. All procedures involving human participants were performed only after approval by the institutional ethics committee. Preparatory activities conducted before ethics approval were limited to operational tasks, including planning, participant screening logistics, and scheduling, and did not involve participant data collection. The protocol was approved by the Ethics Committee of Universidad Técnica de Ambato under code 009-CEISH-UTA-2025, with approval granted on May 7, 2025. The investigation adhered to the principles outlined in the Declaration of Helsinki, and all participant information was anonymized before statistical processing and reporting.

The primary dependent variable was total 100 m sprint time. Maximal inspiratory pressure (PImax) and the individualized inspiratory load corresponding to 40% of PImax were used to prescribe the respiratory intervention. The independent variable was the warm-up condition: PMA alone or PMA plus IMA. This structure was appropriate given the modest sample size and the applied nature of the intervention, as acute sprint-performance differences in university populations are often small in absolute magnitude. Comparing each participant against him- or herself improved internal consistency and reduced the influence of inter-individual heterogeneity. The standardized track setting, identical sprint distance, consistent measurement procedure, and participant-specific inspiratory loading contributed to a controlled experimental framework.

This protocol addressed a practical question in sports physiotherapy and exercise science: whether adding a brief inspiratory muscle warm-up to a conventional peripheral warm-up alters sprint performance in a non-elite but physically active university population. This applied focus supports the translational value of the design, particularly in settings where low-cost, non-pharmacological pre-performance interventions are of interest.

### 2.2 Participants

**Scheme 1:**
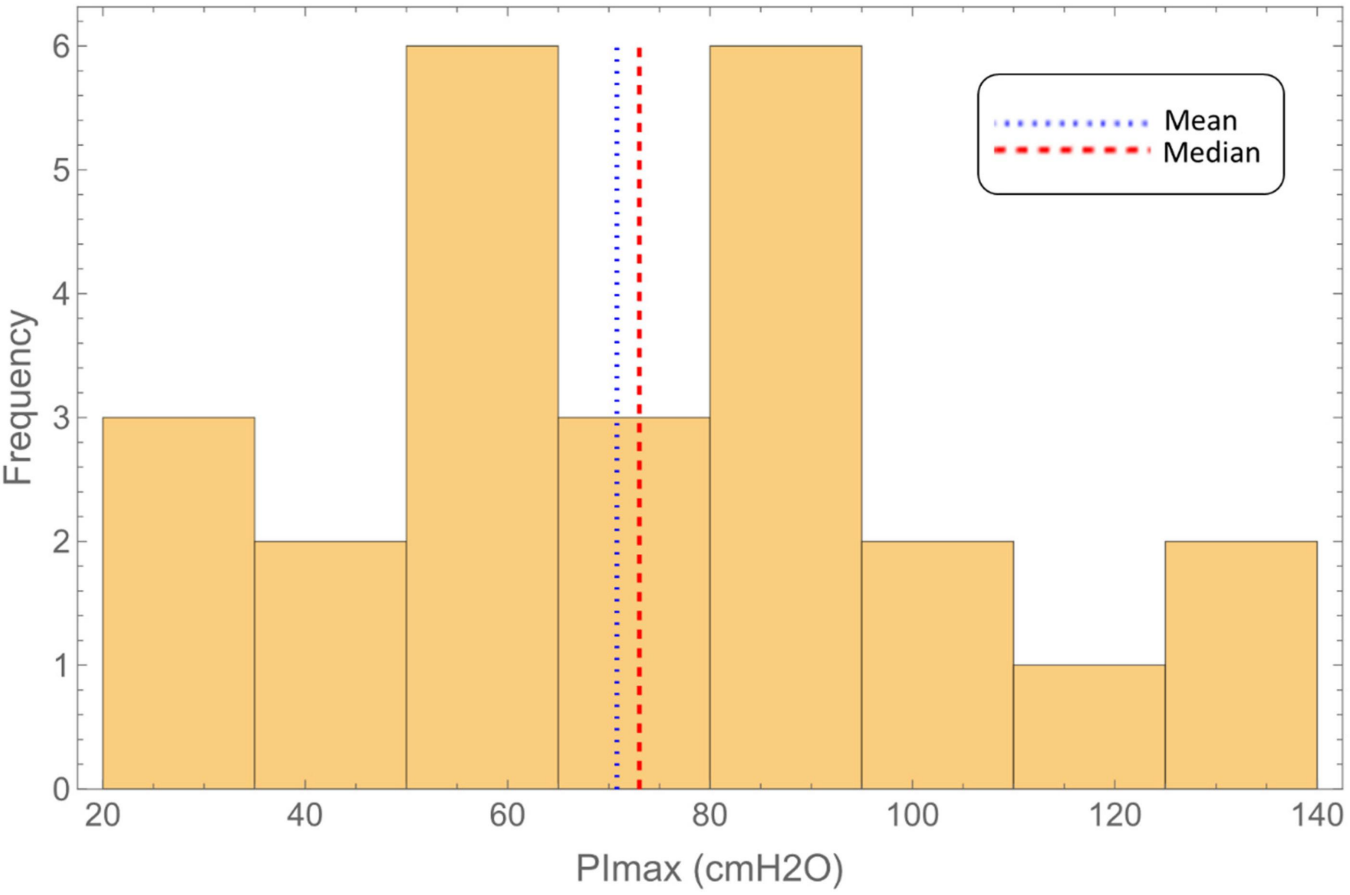
Participant selection and eligibility criteria used in the study.

A convenience sample was recruited from a source population of 136 university students, resulting in a final sample of 25 physically active participants, including 18 women and 7 men, aged 20 to 25 years. All participants were classified as physically active and were considered safe to perform vigorous exercise according to the screening procedures used in the study. Because the sample included both women and men with an unequal sex distribution, the study was not powered to test sex-by-condition effects. Sex-stratified outcomes were therefore examined descriptively to contextualize biological variability, while the primary analysis retained the within-subject comparison between PMA and IMA (Scheme 1).

All participants signed written informed consent before participation. Descriptive characteristics of the sample are shown in Table 1. Participants had a mean age of 21.12 ± 1.20 years, mean body mass of 55.34 ± 6.63 kg, mean height of 1.58 ± 0.06 m, and mean body mass index (BMI) of 22.01 ± 2.15 kg/m^2^. Individual participant data are provided in Supplementary Table S1. Baseline inspiratory strength and the corresponding inspiratory load are also reported in Table 1, as these variables characterize both the sample and the individualized intervention dose.

**Table 1:** Baseline characteristics of the study sample.

Variable	Total sample
Age, years	21.12 ± 1.20
Body mass, kg	55.34 ± 6.63
Height, m	1.58 ± 0.06
BMI, kg/m^2^	22.01 ± 2.15
PImax, cm H2O	73.02 ± 29.88
40% of PImax, cm H2O	29.21 ± 11.95

### 2.3 Pre-Assessment procedures and instrumentation

Anthropometric variables included age, body mass, height, and body mass index (BMI). These measures were recorded before performance testing and were used to characterize the sample and contextualize sprint performance. Given the applied setting and the main objective of the study, anthropometric variables were treated as descriptive measures rather than predictive variables.

The central physiological measurement for intervention prescription was maximal inspiratory pressure (PImax). PImax was assessed before either warm-up protocol and followed the recommendations of the American Thoracic Society (ATS) and European Respiratory Society (ERS). The assessment was performed using a digital manometer. Prior validation work has reported excellent reliability for this type of measurement, with intraclass correlation coefficients of 0.998 for PImax and 0.999 for maximal expiratory pressure (PEmax) when compared with the MicroRPM digital manometer.

The cohort mean PImax was 73.02 ± 29.88 cmH_2_O, and the corresponding mean inspiratory resistance used for the intervention, defined as 40% of PImax, was 29.21 ± 11.95 cmH_2_O. This individualized prescription adjusted respiratory loading to each participant's inspiratory capacity rather than applying a fixed absolute resistance. Descriptive values indicated lower PImax values in women than in men, supporting the use of participant-specific loading across the mixed-sex sample.

Sprint time was measured using the MySprint mobile application, version 2.0.1, selected for its practicality in field-based sprint assessment and its previously reported reliability and validity for sprint performance analysis. The inspiratory intervention was delivered using a POWERbreathe® inspiratory muscle training device (POWERbreathe International Ltd., Southam, Warwickshire, UK), which allowed resistance to be set according to each participant's 40% PImax threshold. The use of a commercially available threshold-loading device supports protocol reproducibility in sports and rehabilitation settings.

### 2.4 Warm-up protocols and inspiratory load prescription

**Scheme 2:**
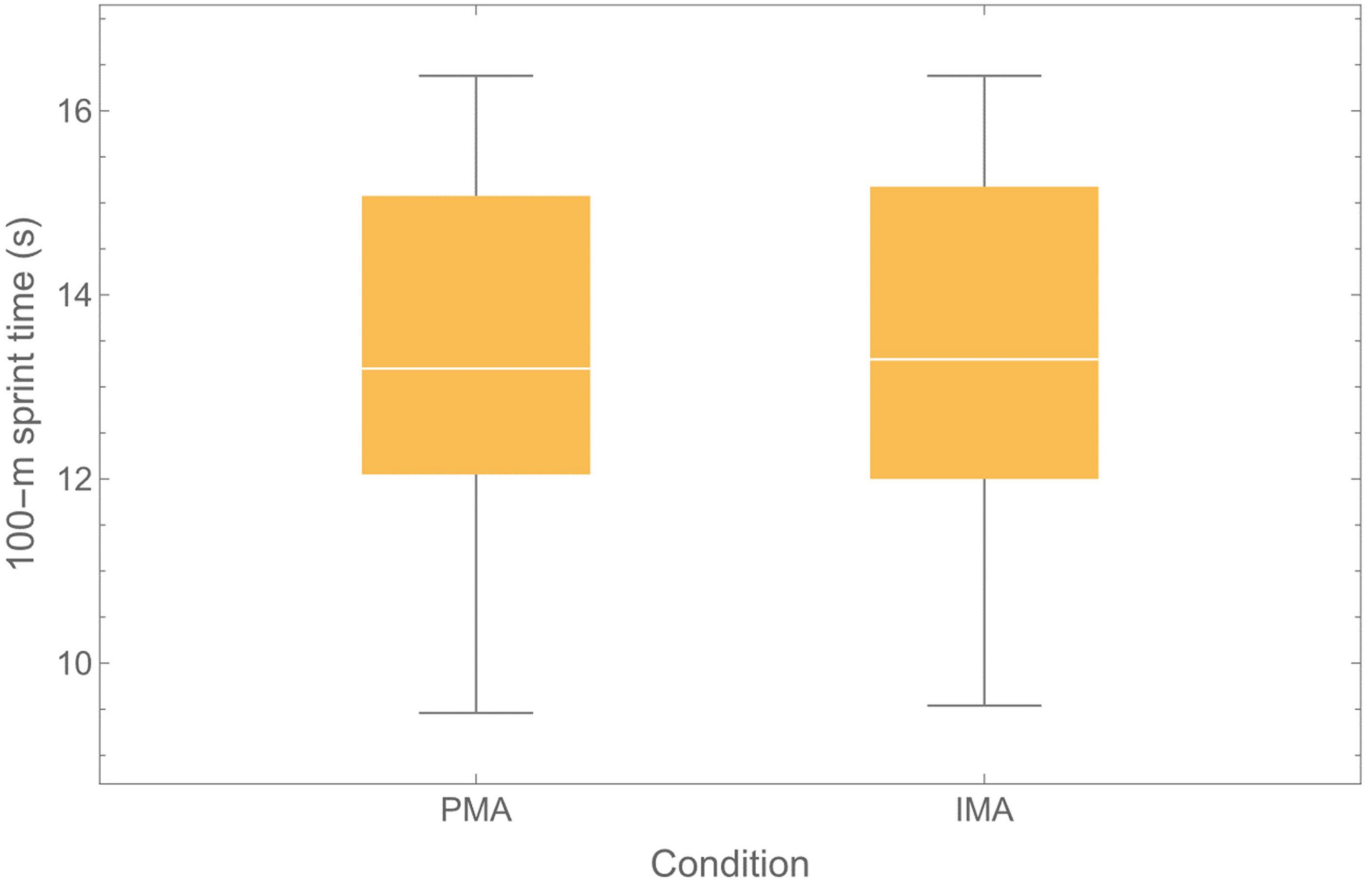
The PMA warm-up protocol applied before sprint testing

Two warm-up conditions were compared: PMA and IMA. Both conditions were applied to the same participants in separate intervention sessions, allowing direct within-subject comparison of performance outcomes. The PMA protocol represented the conventional warm-up condition, whereas the IMA protocol consisted of the same peripheral warm-up plus an additional inspiratory loading component. Therefore, the IMA condition was interpreted as an incremental warm-up condition rather than as an alternative warm-up replacing PMA (Scheme 2).

**Scheme 3:**
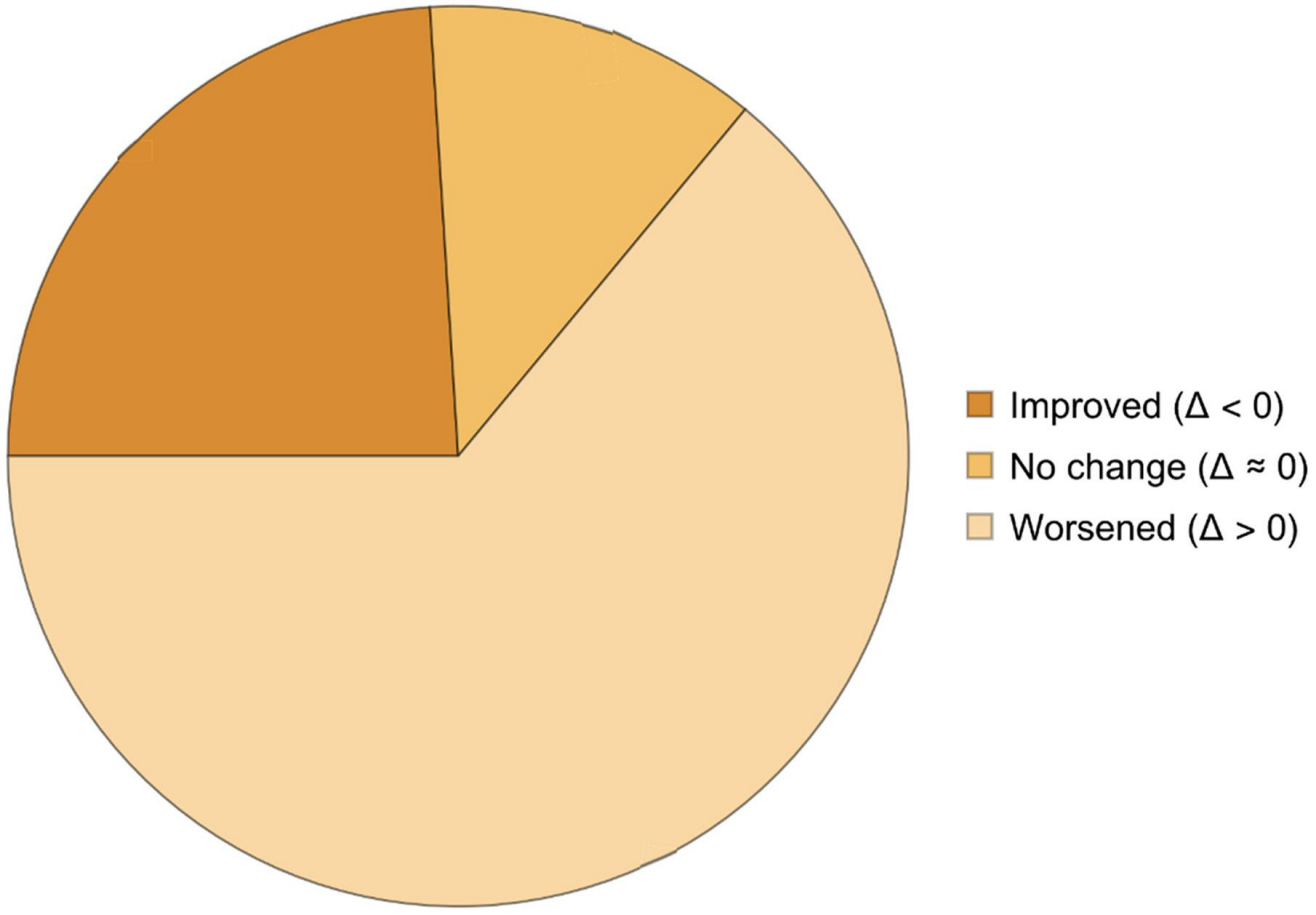
The IMA protocol applied prior to sprint testing.

The PMA protocol lasted 10 min and consisted of two parts. First, participants performed 5 min of moderate-intensity aerobic activity, including 3 min of forward jogging, 1 min of lateral stepping, and 1 min of backward jogging. Second, they completed 5 min of dynamic stretching targeting five lower-limb muscle groups: quadriceps, hamstrings, hip flexors, hip adductors, and gluteal muscles. Each dynamic stretch was performed for 30 s per limb. This structure combined temperature elevation, joint mobilization, and task-relevant neuromuscular activation before sprint testing (Scheme 3).

The IMA protocol included the entire PMA sequence followed by a respiratory-specific warm-up using a POWERbreathe® device. Inspiratory resistance was set at 40% of each participant's PImax, and the protocol consisted of two sets of 15 resisted inspirations, with 1 min of rest between sets. In the present sample, this corresponded to an average inspiratory resistance of 29.21 ± 11.95 cmH_2_O. Relative load prescription was used to scale respiratory demand according to each participant's inspiratory capacity. The components of both warm-up conditions are summarized in Table 2.

**Table 2:** Summary of experimental warm-up conditions.

Condition	Component	Description
PMA	Aerobic activity	3 min forward jogging, 1 min lateral steps, 1 min backward jogging
PMA	Dynamic stretching	5 lower-limb stretches, 30 s per limb: quadriceps, hamstrings, hip flexors, adductors, gluteal muscles
IMA	PMA	Entire PMA protocol completed first
IMA	Inspiratory loading	POWERbreathe®, 2 sets × 15 breaths at 40% PImax, 1-min rest between sets

### 2.5 Sprint testing procedures and outcome measures

Immediately after completing either warm-up protocol, participants performed one maximal 100 m sprint in accordance with International Association of Athletics Federations regulations. The 100 m sprint was selected as the primary performance test because it represents a short-duration, high-intensity task in which any acute effect of a warm-up intervention would need to occur within a brief time frame. The primary outcome was total sprint time, recorded in seconds using the MySprint mobile application.

The same measurement procedure, device, and track environment were used across both experimental conditions to improve consistency between sessions. MySprint was selected because it has been reported as a valid and reliable field-based tool for sprint analysis, with an intraclass correlation coefficient of 1.00 and coefficients of variation between 0.03% and 0.14%. Sprint time was used because it is an easily interpretable field-based performance outcome for coaches, athletes, and clinicians working in sprint or speed-training contexts. No secondary physiological outcomes, such as heart rate, blood lactate, respiratory frequency, oxygen uptake, or perceived dyspnea, were collected during or after the sprint.

### 2.6 Statistical analysis

Statistical analyses were performed using IBM SPSS Statistics. Data distribution was assessed using the Shapiro–Wilk test. Because the paired differences between PMA and IMA were not normally distributed, the Wilcoxon signed-rank test was used to compare 100 m sprint time between conditions. Statistical significance was set at *p* ≤ 0.05.

To complement the inferential analysis, the magnitude of the response was described using absolute and percentage change. Absolute change was calculated as IMA−PMA, with positive values indicating slower sprint performance after IMA. Percentage change was calculated for each participant as [(IMA−PMA)/PMA] × 100. These values were used to evaluate the practical magnitude of the intervention effect beyond statistical significance.

## 3 Results

### 3.1 Baseline physiological and anthropometric profile

The cohort consisted of 25 physically active university students (18 women and 7 men) with a relatively homogeneous age distribution (21.12 ± 1.20 years). Anthropometric data showed a generally lean profile. Mean body mass was 55.34 ± 6.63 kg and mean height was 1.58 ± 0.06 m, resulting in an average body mass index (BMI) of 22.01 ± 2.15 kg/m^2^. Individual data (Supplementary Table S1) showed moderate dispersion, with BMI values ranging from 17.72 to 27.06 kg/m^2^, indicating variability in body size across participants. This variability is relevant for contextualizing performance outcomes, as body composition may influence sprint mechanics and force production.

**Figure 1:**
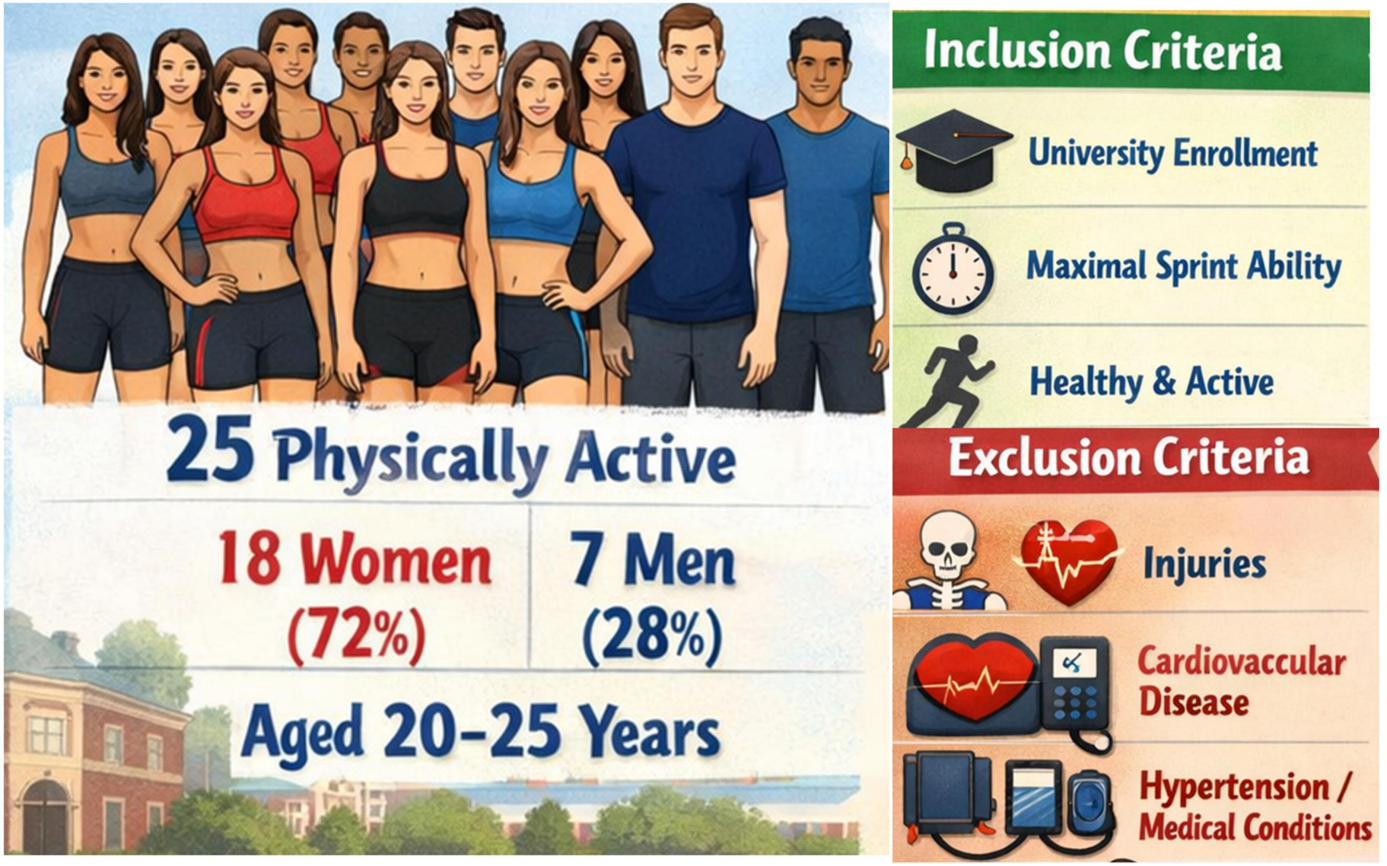
Distribution of maximal inspiratory pressure (PImax) values in the study sample.

Inspiratory muscle strength exhibited substantial inter-individual variability. Mean PImax was 73.02 ± 29.88 cmH_2_O, with a range from 24.30 to 139.00 cmH_2_O (Supplementary Table S3). This heterogeneity is represented in Figure 1, where PImax values show a broad spread across participants. The proximity between the mean (73.02 cmH_2_O) and median (70.80 cmH_2_O) suggests a relatively symmetric central tendency despite the wide dispersion. Descriptive inspection of Supplementary Table S1 indicated a tendency toward higher PImax values in men than in women; however, sex-based comparisons were not a primary objective of the study. The inspiratory load prescribed for the intervention, defined as 40% of PImax, averaged 29.21 ± 11.95 cmH_2_O and ranged from 9.72 to 55.60 cmH_2_O. The wide distribution of these values reflects the use of individualized relative-load prescription, ensuring that the respiratory stimulus was scaled according to each participant's inspiratory capacity.

### 3.2 Sprint performance outcomes and between-condition comparison

**Figure 2:**
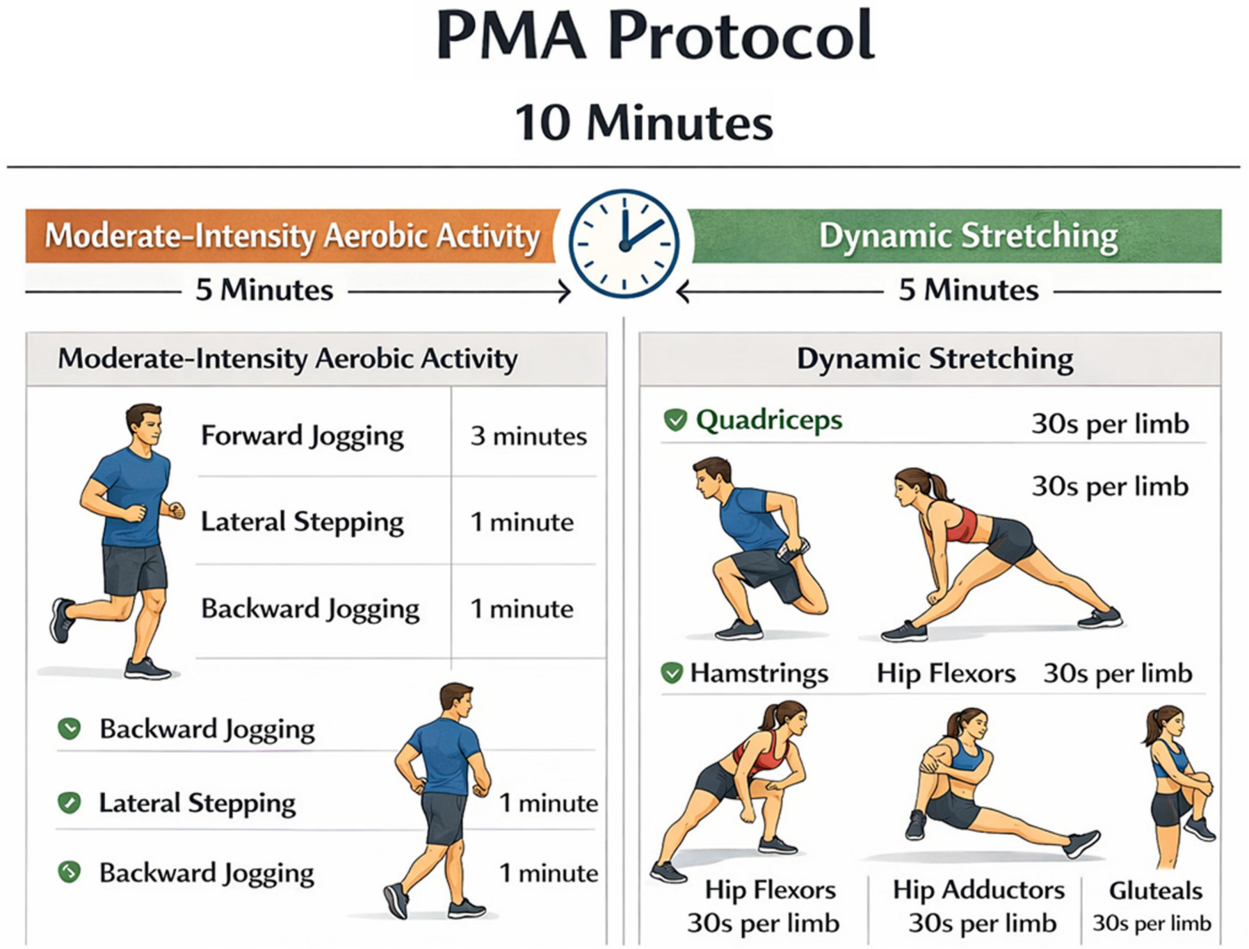
Comparison of 100 m sprint performance between PMA and IMA conditions.

Sprint performance outcomes under both warm-up conditions are presented in Supplementary Table S2 and summarized graphically in Figure 2. The mean 100 m sprint time following the peripheral muscle activation (PMA) protocol was 12.92 ± 2.12 s, while the IMA condition resulted in a mean time of 12.96 ± 2.10 s. These values indicate negligible differences between conditions at the group level. As shown in Figure 2, the distribution of sprint times was highly similar between conditions. The boxplots reveal overlapping interquartile ranges and comparable medians, indicating that central tendency and dispersion were consistent across both protocols. The spread of values, approximately 9.50 to 16.40 s, was nearly identical in PMA and IMA, further supporting the absence of a meaningful shift in performance distribution following inspiratory pre-activation.

Sex-specific descriptive analysis showed a similar pattern. Women recorded mean sprint times of 12.82 ± 1.99 s after PMA and 12.83 ± 1.95 s after IMA, while men recorded 13.17 ± 2.57 s after PMA and 13.29 ± 2.59 s after IMA. These values showed trivial between-condition changes in both subgroups and were used only to contextualize biological variability rather than to infer sex-specific effects. Analysis of paired differences between IMA and PMA yielded a mean absolute change of 0.039 ± 0.145 s, corresponding to an approximate percentage change of 0.30% relative to PMA. Individual absolute changes ranged from −0.240 to 0.620 s (see Supplementary Table S3), indicating heterogeneous but small within-subject variations. Normality testing revealed a non-normal distribution of paired differences (Shapiro–Wilk W = 0.595, *p* < 0.001), supporting the use of non-parametric testing. The Wilcoxon signed-rank test detected a statistically significant difference between conditions (W = 43.0, *p* = 0.0038); however, the magnitude of this difference was trivial and unlikely to be practically meaningful.

Sprint performance was highly consistent between conditions (r = 0.998, *p* < 0.001; Supplementary Table S5), indicating strong within-subject reproducibility regardless of the warm-up protocol. No meaningful correlations were observed between PImax and sprint time under either condition (PMA: r = −0.127, *p* = 0.546; IMA: r = −0.103, *p* = 0.626), suggesting that baseline inspiratory muscle strength was not associated with sprint performance in this cohort.

### 3.3 Individual variability and response patterns

As shown in Supplementary Table S3, the mean paired change was 0.039 ± 0.145 s, with individual values ranging from −0.240 to 0.620 s. These values indicate heterogeneous responses to the inspiratory intervention. Some participants showed small improvements in sprint performance after IMA, whereas others showed trivial changes or slight impairments, suggesting the absence of a uniform response.

**Figure 3:**
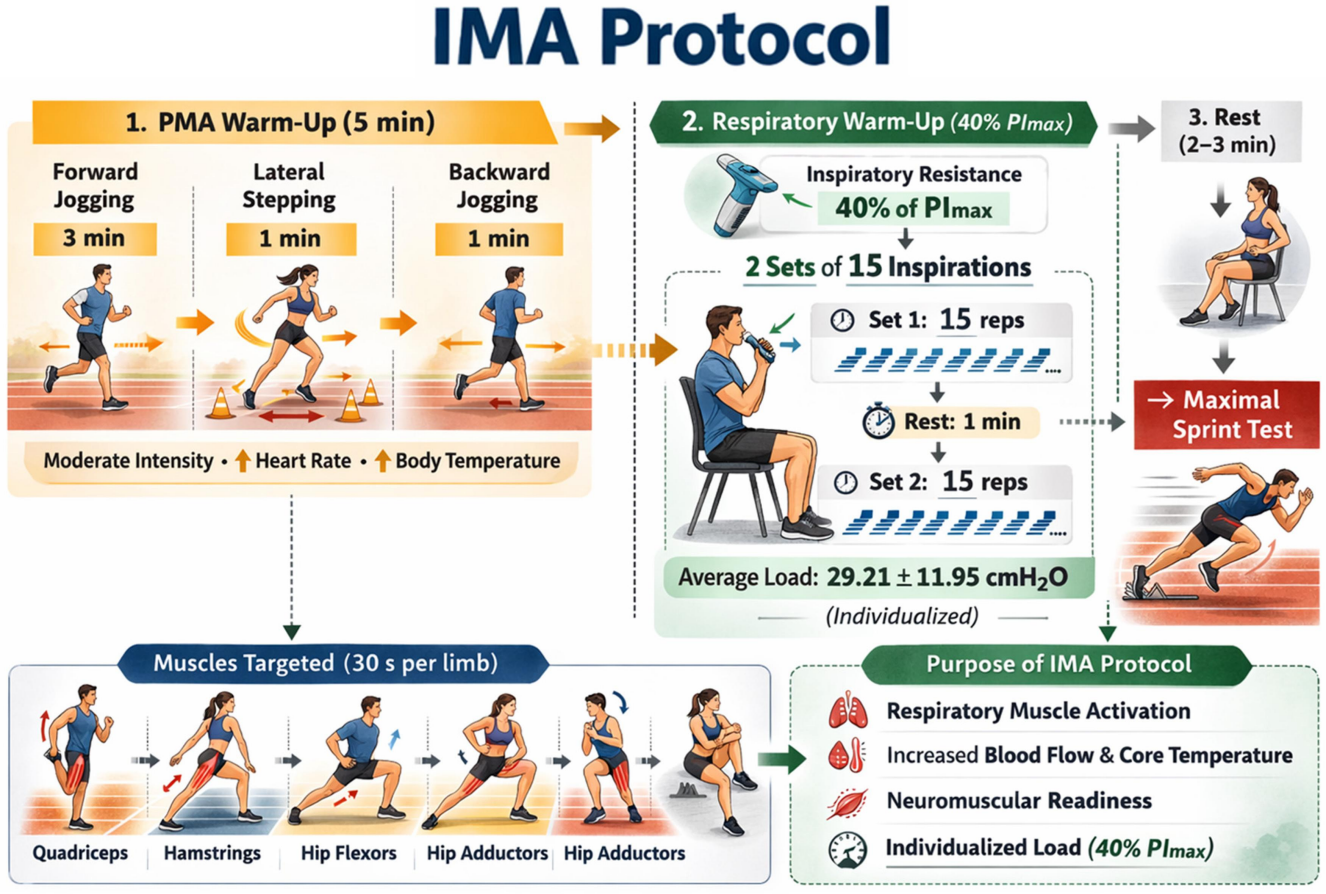
Distribution of individual response patterns after inspiratory muscle pre-activation relative to peripheral muscle activation alone.

This variability is illustrated in Figure 3, which presents individual response categories as improved, unchanged, or worsened performance after inspiratory pre-activation. The largest proportion of participants fell within the worsened category, whereas fewer participants showed improvement and only a small number showed no change. Despite this distribution, the absolute magnitude of the changes remained very small, indicating that the observed differences were not practically meaningful in performance terms. The descriptive pattern shown in Figure 3 is consistent with the inferential results reported in Supplementary Table S4. Although the Wilcoxon signed-rank test indicated a statistically significant difference between PMA and IMA (W = 43.0, *p* = 0.0038), the average change was less than 0.05 s, which is unlikely to represent a meaningful ergogenic effect in applied sprint settings. The non-normal distribution of paired differences identified by the Shapiro–Wilk test (W = 0.595, *p* < 0.001) further supports the interpretation that responses were irregularly distributed rather than consistently favoring one warm-up condition.

Additional support for the stability of individual performance is provided in Supplementary Table S5, where sprint times under PMA and IMA showed an almost perfect association (r = 0.998, *p* < 0.001). This strong correspondence indicates that participant ranking remained essentially unchanged across conditions, despite small within-subject fluctuations. Likewise, the absence of significant correlations between PImax and sprint time under either protocol suggests that baseline inspiratory strength was not associated with the direction or magnitude of the acute performance response.

## 4 Discussion

The main finding was that adding inspiratory loading to a conventional warm-up did not produce a practically meaningful improvement in 100 m sprint performance. Although a statistically significant difference was detected between conditions, the mean absolute change was only 0.039 s, corresponding to an approximate percentage change of 0.30% relative to PMA. This magnitude was too small to support a performance-enhancing effect in this context. The result is consistent with the physiological characteristics of the 100 m sprint, where performance is determined mainly by rate of force development, motor unit recruitment, sprint mechanics, and mechanical efficiency rather than by respiratory limitations. Given the short duration of the event and its predominant reliance on phosphagen metabolism, interventions targeting inspiratory muscle function may have limited capacity to influence a single maximal sprint effort (7).

Sex-stratified descriptive results were consistent with the pooled analysis, with trivial between-condition changes in both women and men. The unequal subgroup sizes prevented assessment of sex-by-condition effects, and biological variability related to anthropometry, inspiratory strength, and sprint mechanics may have contributed to the heterogeneous individual responses. The within-subject design reduced the influence of stable individual differences, but it did not remove this source of variability.

Previous research on inspiratory muscle warm-up has reported mixed findings across exercise modalities. Positive effects have been observed in endurance-based activities, such as distance running, rowing, and cycling, where respiratory demand is sustained and the work of breathing may become limiting (54, 55). In short-duration or high-intensity efforts, effects appear less consistent. Minahan et al. (56) found that repeated-sprint cycling did not induce respiratory muscle fatigue, while Wüthrich et al. (57) reported no meaningful improvement in muscle contractility after acute respiratory loading. These findings support a task-specific interpretation of inspiratory muscle pre-activation. Mechanisms such as attenuation of the inspiratory muscle metaboreflex may be relevant during prolonged or repeated high-intensity exercise, but they are unlikely to have a major influence during a single 100 m sprint (2).

A mechanical role of the inspiratory muscles remains plausible because the diaphragm contributes to intra-abdominal pressure, trunk stiffness, and force transmission during maximal running. In the present study, any acute enhancement in inspiratory muscle activation was either too small to affect sprint time or was overshadowed by dominant neuromuscular determinants of sprint performance. Individual responses also varied, with some participants showing slight improvements and others slight decrements. These changes were small and inconsistent, reinforcing the absence of a systematic performance effect. The lack of association between PImax and sprint time under either condition indicates that baseline inspiratory strength was not a key determinant of acute sprint performance in this cohort (58).

From a practical perspective, the present findings do not support inspiratory muscle pre-activation as a priority strategy for improving 100 m sprint performance. Although no relevant performance impairment was observed, its use should be considered complementary and secondary to warm-up components that directly target neuromuscular readiness, sprint mechanics, and muscle temperature. This interpretation is consistent with recent evidence indicating that inspiratory muscle warm-up effects are task-dependent and are more likely to emerge when respiratory demand is sustained or repeated rather than during a single brief maximal effort (35, 54–58).

## 5 Limitations

Several limitations should be considered when interpreting these findings. The sample included physically active university students rather than trained sprinters or elite athletes, which limits the generalizability of the results to competitive sprint populations. Because the 100 m sprint is a technically demanding event, limited sprint-specific experience may have increased performance variability and reduced the ability to detect small intervention effects. The inclusion of both women and men also introduces biological variability related to anthropometry, inspiratory muscle strength, sprint mechanics, and neuromuscular profile. Although the within-subject design reduced inter-individual variability by comparing each participant under both warm-up conditions, the sample size and unequal sex distribution did not allow adequately powered sex-based inferential analysis. For this reason, sex-stratified values were interpreted descriptively rather than as independent subgroup effects.

The study also evaluated only the acute response to a single inspiratory muscle pre-activation protocol. Therefore, the findings should not be extrapolated to chronic inspiratory muscle training or to repeated use of this strategy across several training sessions. The protocol used a fixed relative load of 40% PImax and two sets of 15 resisted inspirations; different loads, volumes, or recovery intervals may produce different responses.

Another limitation is the absence of secondary physiological measures during or after the sprint, such as heart rate, blood lactate, respiratory frequency, oxygen uptake, or perceived dyspnea. As a result, the interpretation is restricted to external sprint performance and inspiratory strength-based load prescription. This limits mechanistic conclusions about metabolic demand, respiratory muscle fatigue, or autonomic responses. External factors such as prior recovery, nutrition, motivation, sleep, and day-to-day variability in sprint execution may also have influenced individual responses, despite the repeated-measures design.

## 6 Conclusions

The present study investigated the acute effects of adding inspiratory muscle pre-activation to a conventional warm-up protocol on 100 m sprint performance in physically active university students. Although a statistically significant difference was observed between conditions, the mean absolute change was small (0.039 s; approximately 0.30% relative to PMA) and did not support a practically meaningful improvement in sprint performance. These findings indicate that, in the context of a single short-duration maximal sprint, inspiratory muscle pre-activation should not be interpreted as an ergogenic strategy for improving 100 m performance.

This interpretation is consistent with the physiological demands of the 100 m sprint, where performance is driven mainly by neuromuscular readiness, rapid force production, sprint mechanics, and mechanical efficiency rather than by respiratory limitations. The observed inter-individual variability suggests that responses to inspiratory interventions may differ across participants, but the present sample size and mixed-sex composition limit stronger subgroup conclusions.

From an applied perspective, inspiratory muscle pre-activation may be included within a broader warm-up routine, but the present data do not support its use as a primary strategy to improve 100 m sprint performance. Practitioners should prioritize established warm-up components that directly target neuromuscular activation, sprint-specific movement preparation, and muscle temperature.

## Data Availability

The original contributions presented in the study are included in the article/Supplementary Material, further inquiries can be directed to the corresponding author.
