# Supplementary material for: Effects of inspiratory muscle Pre-activation on 100 m sprint performance in physically active university students
Source: Front Sports Act Living. 2026 Jul 20;8:1840117. doi: 10.3389/fspor.2026.1840117 (PMC13429728; doi:10.3389/fspor.2026.1840117)
Supplement: Supplementary file 1 [file Table1.docx]

**SUPPLEMENTARY INFORMATION**

**Effects of Inspiratory Muscle Preactivation on 100-m Sprint Performance in Physically Active University Students**

Javier Carrión ^1, ,⊺,^*, Stalin Javier Caiza Lema ^2,⊺^, Samira Anahí Masaquiza Sanguil ^2^, Maria Augusta Latta Sánchez ^2^, Andrea Carolina Peñafiel Luna ^2^, Paúl Adrián Arias Córdova ^2^, Angela Priscila Campos Moposita ^2^,Josselyn Gabriela Bonilla Ayala ^2^ ,Martha Montalvan^3^, Raynier Zambrano-Villacrés ^4,5^*

^1^Department of Chemistry, Universidad Técnica Particular de Loja, Loja, 110160, Ecuador

^2^Carrera de Fisioterapia, Facultad Ciencias de la Salud, Universidad Técnica de Ambato, Ecuador

^3^Escuela de Medicina, Universidad Espíritu Santo, Samborondón 0901952, Ecuador marielandrade@uees.edu.ec

^4^Facultad de Ciencias de la Salud y Desarrollo Humano, Universidad ECOTEC, Km.13.5 Samborondón, Samborondón, EC092302, Ecuador

^5^Doctorado en Ciencias Biomédicas, Escuela de Medicina y Ciencias de la Salud, Instituto Universitario Italiano de Rosario (IUNIR), Rosario S2000CTT, Argentina

^⊺^These authors contribute equally

***Correspondence:** razambrano@ecotec.edu.ec (R.Z-V)

**Table S1.** Individual anthropometric characteristics, maximal inspiratory pressure (PImax), and prescribed inspiratory load (40% PImax) of the study participants (n = 25).

| **Code** | **Age (Years)** | **Body mass (Kg)** | **Height**  **(m)** | **BMI**  **(kg/m²)** | **PImax**  **cm H2O** | **40% of PImax**  **cm H2O** |
| --- | --- | --- | --- | --- | --- | --- |
| F1 | 20 | 53 | 1.58 | 21.23 | 91.60 | 36.64 |
| F2 | 23 | 57.5 | 1.57 | 23.33 | 58.70 | 23.48 |
| F3 | 20 | 50 | 1.53 | 21.36 | 60.30 | 24.12 |
| F4 | 20 | 53 | 1.52 | 22.94 | 50.20 | 20.08 |
| F5 | 22 | 56.24 | 1.6 | 21.97 | 139.00 | 55.60 |
| F6 | 22 | 64 | 1.54 | 26.99 | 86.00 | 34.40 |
| F7 | 21 | 49 | 1.48 | 22.37 | 57.00 | 22.80 |
| F8 | 21 | 50 | 1.55 | 20.81 | 105.00 | 42.00 |
| F9 | 22 | 52 | 1.6 | 20.31 | 44.20 | 17.68 |
| F10 | 22 | 65 | 1.65 | 23.88 | 24.30 | 9.72 |
| F11 | 20 | 54 | 1.62 | 20.58 | 61.30 | 24.52 |
| F12 | 20 | 44.4 | 1.54 | 18.72 | 111.30 | 44.52 |
| F13 | 21 | 51 | 1.55 | 21.23 | 90.10 | 36.04 |
| F14 | 21 | 49 | 1.5 | 21.78 | 70.00 | 28.00 |
| F15 | 20 | 56.5 | 1.57 | 22.92 | 96.20 | 38.48 |
| F16 | 21 | 64 | 1.63 | 24.09 | 70.80 | 28.32 |
| F17 | 21 | 65 | 1.55 | 27.06 | 32.00 | 12.80 |
| F18 | 21 | 49 | 1.5 | 21.78 | 26.90 | 10.76 |
| M1 | 20 | 60 | 1.67 | 21.51 | 81.00 | 32.40 |
| M2 | 21 | 58 | 1.6 | 22.66 | 58.00 | 23.20 |
| M3 | 22 | 60 | 1.7 | 20.76 | 81.90 | 32.76 |
| M4 | 22 | 44.8 | 1.59 | 17.72 | 125.20 | 50.08 |
| M5 | 25 | 65 | 1.68 | 23.03 | 93.90 | 37.56 |
| M6 | 20 | 49 | 1.6 | 19.14 | 35.50 | 14.20 |
| M7 | 20 | 64 | 1.7 | 22.15 | 75.10 | 30.04 |

*F(n) (Feminine); M(n) (Masculine)

**Table S2.** Descriptive comparison of 100-m sprint performance after peripheral muscle activation (PMA) and inspiratory muscle activation (IMA) in the total sample and by sex.

| **Outcome** | **Total sample** | **Women** | **Men** |
| --- | --- | --- | --- |
| Sprint time after PMA, s | 12.92 ± 2.12 | 12.82 ± 1.99 | 13.17 ± 2.57 |
| Sprint time after IMA, s | 12.96 ± 2.10 | 12.83 ± 1.95 | 13.29 ± 2.59 |

**Table S3.** Descriptive statistics of anthropometric variables, inspiratory muscle strength, and sprint performance outcomes under both warm-up conditions

| **Variable** | **Mean ± SD** | **Median** | **Minimum** | **Maximum** |
| --- | --- | --- | --- | --- |
| Age (Years) | 21.12 ± 1.20 | 21.00 | 20.00 | 25.00 |
| Weight (kg) | 55.34 ± 6.63 | 53.00 | 44.40 | 65.00 |
| PImax (cmH₂O) | 73.02 ± 29.88 | 70.80 | 24.30 | 139.00 |
| 40% PImax (cmH₂O) | 29.21 ± 11.95 | 28.32 | 9.72 | 55.60 |
| 100-m sprint, peripheral warm-up (s) | 12.92 ± 2.12 | 12.92 | 9.46 | 16.38 |
| 100-m sprint, inspiratory preactivation (s) | 12.96 ± 2.10 | 12.96 | 9.54 | 16.38 |
| Paired difference (Insp − Periph) (s) | 0.039 ± 0.145 | 0.030 | -0.240 | 0.620 |

**Table S4.** Statistical comparison between peripheral muscle activation and inspiratory muscle activation conditions, including parametric and non-parametric tests and normality assessment.

| **Analysis** | **Statistic** | **p value** |
| --- | --- | --- |
| Paired t-test: peripheral vs inspiratory | 1.348 | 0.190 |
| Wilcoxon signed-rank test | 43.0 | 0.0038 |
| Shapiro-Wilk test for paired differences | 0.595 | < 0.001 |

**Table S5.** Correlation analysis between PImax and sprint performance, as well as between warm-up conditions.

| **Variables compared** | **Correlation coefficient (r)** | **p value** |
| --- | --- | --- |
| PImax vs peripheral sprint time | - 0.127 | 0.546 |
| PImax vs inspiratory sprint time | - 0.103 | 0.626 |
| Peripheral vs inspiratory sprint time | 0.998 | < 0.001 |
